# The Role of Urinary Extracellular Vesicles Sodium Chloride Cotransporter in Subtyping Primary Aldosteronism

**DOI:** 10.3389/fendo.2022.834409

**Published:** 2022-04-04

**Authors:** Linghui Kong, Xiaofeng Tang, Yuanyuan Kang, Lei Dong, Jianhua Tong, Jianzhong Xu, Ping-jin Gao, Ji-guang Wang, Weili Shen, Limin Zhu

**Affiliations:** ^1^ Department of Cardiovascular Medicine, State Key Laboratory of Medical Genomics, Shanghai Key Laboratory of Hypertension, Shanghai Institute of Hypertension, Ruijin Hospital, Shanghai Jiao Tong University School of Medicine, Shanghai, China; ^2^ Department of Pathology, Ruijin Hospital, Shanghai Jiaotong University School of Medicine, Shanghai, China; ^3^ Department of Laboratory Medicine and Central Laboratory, Ruijin Hospital, Shanghai Jiao Tong University School of Medicine, Shanghai, China

**Keywords:** primary aldosteronism, adrenal venous sampling, extracellular vesicles, NCC, *KCNJ5*

## Abstract

**Background:**

Adrenal venous sampling (AVS) is recognized as the gold standard for subtyping primary aldosteronism (PA), but its invasive nature and technical challenges limit its availability. A recent study reported that sodium chloride cotransporter (NCC) in urinary extracellular vesicles (uEVs) is a promising marker for assessing the biological activity of aldosterone and can be treated as a potential biomarker of PA. The current study was conducted to verify the hypothesis that the expression of NCC and its phosphorylated form (pNCC) in uEVs are different in various subtypes and genotypes of PA and can be used to select AVS candidates.

**Methods:**

A total of 50 patients with PA were enrolled in the study. Urinary extracellular vesicles (uEVs) were isolated from spot urine samples using ultracentrifugation. NCC and pNCC expressions were tested in patients diagnosed with PA who underwent AVS. Sanger sequencing of *KCNJ5* was performed on DNA extracted from adrenal adenoma.

**Results:**

pNCC (1.89 folds, *P*<.0001) and NCC (1.82 folds, *P*=0.0002) was more abundant in the uEVs in the high lateralization index (h-LI, ≥ 4) group than in the low LI (l-LI, < 4) group. Carriers of the somatic *KCNJ5* mutations, compared with non-carriers, had more abundant pNCC expression (2.16 folds, P=0.0039). Positive correlation between pNCC abundance and plasma aldosterone level was found in this study (R = 0.1220, *P* = 0.0129).

**Conclusions:**

The expression of pNCC in uEVs in patients with PA with various subtypes and genotypes was different. It can be used as biomarker of AVS for PA subtyping.

## Introduction

Primary aldosteronism (PA) is the most common form of secondary hypertension, characterized by aldosterone overproduction and suppression of plasma renin activity (1, 2). Patients with unilateral aldosteronism confirmed by adrenal venous sampling (AVS) can be treated with adrenalectomy. Those with bilateral hyperaldosteronism are treated mainly with mineralocorticoid receptor antagonists. More than 30% of operated patients have clinical success, and 94% have biochemical success according to the PASO study (3). Potassium inwardly-rectifying channel subfamily J member 5 (*KCNJ5*) was the first identified gene mutated in aldosterone-producing adenoma (APA) (4). Recent studies have suggested that *KCNJ5* mutation is a protective factor for complete clinical success (5–7). To date, AVS is generally regarded as the gold standard test for distinguishing between unilateral and bilateral aldosteronism; the lateralization rate has been reported as 50-60%, but it is not widely available due to technical difficulties, cost, and the invasive nature of operation (8–10). Therefore, it is necessary to find alternative criteria or biomarkers that can predict lateralization and accordingly decrease the number of AVS candidates.

The sodium chloride cotransporter (NCC), expressed in the proximal part of the convoluted tubule, is involved in blood pressure regulation by mediating the reabsorption of filtered sodium and water (11). Its function is highly regulated by aldosterone *via* phosphorylation (12). To explore the expression of NCC and phosphorylated NCC (pNCC) *in vivo* in human renal tubules, urinary extracellular vesicles (uEVs) are an ideal non-invasive diagnostic tool compared to renal biopsy. Urinary extracellular vesicles (EVs) are lipid bilayer vesicles, which are released into the urine by cells from all nephron segments *via* exocytosis (13). They are enriched in a variety of proteins, nucleic acids, and lipids. These biomolecules reflect the pathophysiological status of parental cells and can be used as disease biomarkers (14, 15). Recently, Van der Lubbe et al. reported that pNCC in uEVs are promising markers for assessing the biological activity of aldosterone and potential clinical biomarkers for PA (16). Nevertheless, the relationship between NCC or pNCC expression and subtypes of PA has not been clarified.

In light of the above information, the present study was conducted to explore the hypothesis that NCC and pNCC expression in uEVs are different in different PA subtypes or genotypes, and uEVs could be a non-invasive predictive tool to decrease the number of AVS candidates.

## Methods

### Patients

We recruited hospitalized patients who were diagnosed with PA between September 2020 and September 2021 in the Hypertension Department (Ruijin Hospital, Shanghai Jiao Tong University School of Medicine). The workup for PA complied with the 2016 Endocrine Society clinical guidelines for the diagnosis and management of PA, as reported in our previous study (10). In brief, before workup, patients were advised to withdraw mineralocorticoid receptor antagonists for at least 6 weeks, non-potassium sparing diuretics for 4 weeks, and β-blockers, angiotensin-converting enzyme inhibitors, and angiotensin II type 1 receptor blockers (ARBs) for 2 weeks. Non-dihydropyridine Ca^2+^ blockers and/or α_1_-blockers were prescribed for blood pressure (BP) control as necessary. Patients with an aldosterone-renin ratio (ARR) of > 30 (ng/dL)/(ng/mL/h) at least twice at the outpatient clinic were considered as PA candidates and were then admitted to the hospital for a confirmatory test. Patients with hypokalemia were corrected with an oral potassium chloride supplement to reach a serum potassium level of > 3.5 mmol/L. A supine saline infusion test was performed. Patients with post-test plasma aldosterone concentration (PAC) of > 10 ng/dL were diagnosed with PA. All patients underwent thin-sliced (3 mm) adrenal computed tomography (CT). AVS was performed in all patients who were willing to undergo unilateral adrenalectomy. A selectivity index (adrenal cortisol to peripheral cortisol) of ≥ 2.0 was considered correct catheterization and a lateralization index (LI, aldosterone/cortisol ratio from high to low side) of ≥ 4.0 without cosyntropin stimulation was regarded as lateralization. Patients with LI ≥ 4 and < 4 were classified into high LI (h-LI) and low LI (l-LI) groups, respectively. Patients with LI > 4 underwent unilateral adrenalectomy, as well as those with 3 ≤ LI < 4 and a contralateral suppression index of < 1. The remaining patients received medical treatment, mainly mineralocorticoid antagonists. APAs were confirmed using hematoxylin and eosin and *CYP11B2* staining.

### Urinary EVs Study

#### Urine Collection and uEVs Isolation

Briefly, approximately 50 ml first-void morning urine sample was collected during the day of the saline infusion test. Urine sample (10 ml of urine sample was directly used for the measurement of creatinine content using a creatinine [urinary] Assay Kit, 500701-96, Cayman, USA), and the remaining sample was treated with 3 ml PMSF (10 mM) and 50 μl leupeptin (1 mg/ml) before freezing at -80°C (16, 17). Urinary EVs were isolated *via* ultracentrifugation, as previously described by Salih et al. (18). All centrifugations were performed in an ultracentrifuge (Optima XPN-100, Beckman Coulter, USA) with an angle rotor (Type 70 Ti Rotor, Beckman Coulter, USA) at 4°C. Briefly, frozen urine was quickly thawed at 32°C and vortexed for 90 s. The urine was first centrifuged at 17,000×g for 15 min to remove high-density particles. The supernatant obtained above was ultracentrifuged at 200,000×g for 90 min to obtain a gel-like precipitate and the new supernatant was discarded. This gel-like pellet was resuspended in 1× PBS buffer (Sangon Biotech, China), and then ultracentrifuged at 200,000 × g for 90 min to obtain a new pellet containing EVs. The pellet was resuspended in 160 μL 1× PBS buffer and froze at -80°C after aliquoting.

#### Nanoparticle Tracking Analysis

The particle size and concentration of uEVs were measured using nanoparticle tracking analysis (NTA) at VivaCell Biosciences with ZetaView PMX 110 (Particle Metrix, Meerbusch, Germany) and the corresponding software ZetaView 8.04.02. Briefly, uEVs were appropriately diluted in 1× PBS buffer (Biological Industries, Israel) to measure particle size and concentration. After the sample cell was cleaned using 1× PBS buffer (Biological Industries, Israel), the ZetaView system was calibrated using 110 nm polystyrene particles.

#### Transmission Electron Microscopy

The exosomal suspension was dripped into the copper mesh (4406, TEDpella, USA) and left for more than one minute, and then the droplet was negatively stained with 2% phosphotungstic acid solution (G1871, Solarbio, China) and fixed for 10 min. Finally, the samples were analyzed using an electron microscope (Tecnai G2 Spirit BioTWIN, Tecnai, USA) operated at 120 kV.

#### Immunoblotting

The total protein content of uEVs was quantified using the BCA method. Urine creatinine was used for normalization of the samples, as previously described (14, 19). The protein samples were loaded based on the same amount of protein vs creatinine. Protein samples were separated using SDS-PAGE (4 to 12%, Biofuraw™ Precast Bis-Tris Gel), transferred to polyvinylidene difluoride membranes (Bio-Rad Laboratories), and blocked with 5% nonfat milk for 1 h at room temperature. Blots were incubated with primary antibodies against CD9 (1:1000, Abcam, ab92726), CD63 (1:1000, Abcam, ab134045), ALIX (1:1000, Cell Signaling Technology, 2171S), NCC (1:1000, StressMarq, SPC-402), and pNCC (1:1000, PhosphoSolutions, p1311-53) at 4°C overnight. Horseradish peroxidase-conjugated secondary antibodies were diluted in 5% BSA to detect the bound primary antibodies. Immunoreactive bands were detected using ECL reagent (Thermo Scientific, Waltham, MA), and densitometry measurements were performed using Image J (Version 1.53, NIH, USA). CD9 was used as a measure of EVs, and we compared the densitometry of protein vs CD9 between lanes.

### DNA Isolation and Sanger Sequencing of KCNJ5

DNA was obtained from 35 mm tumor slices using Qiagen column separation according to the manufacturer’s instructions (Qiagen, Hilden, Germany). *KCNJ5* sequences were amplified using a previously described primer (20). PCR conditions were as follows: 95°C for 5 min; 35 cycles of 94°C for 1 min, 60°C for 1 min, 72°C for 1 min, and 72°C for 10 min in a 96-well GeneAmp PCR system 9700 (Applied Biosystems) using 20 ng of template DNA. All PCR amplimers were analyzed using 1.2% agarose gel electrophoresis. Sequencing reactions were performed using a BigDye Terminator Cycle Sequencing Kit (Thermo Fisher, Waltham, MA, USA) and analyzed on a 24-capillary 3500 DX DNA Analyzer (Applied Biosystems, USA). Exonic sequences were read and aligned using Chromas software (version 1.62, Technelysium).

### Statistical Analysis

Statistical analyses were performed using SAS software (version 9.4; SAS Institute, Cary, NC, USA) and GraphPad Prism 6.0 (GraphPad Software, Inc., San Diego, CA). Results were expressed as the mean and standard deviation or the median, as appropriate. Data were logarithmically transformed before analysis in the case of a non-normal distribution. Student’s t-test or analysis of variance was used for group comparison. Pearson correlation was used to analyze correlations between different biochemical indicators and urinary sodium transporters (NCC and pNCC). Statistical significance was set at *P*<0.05.

## Results

### Clinical Characteristics in Patients With PA

**Figure 1:**
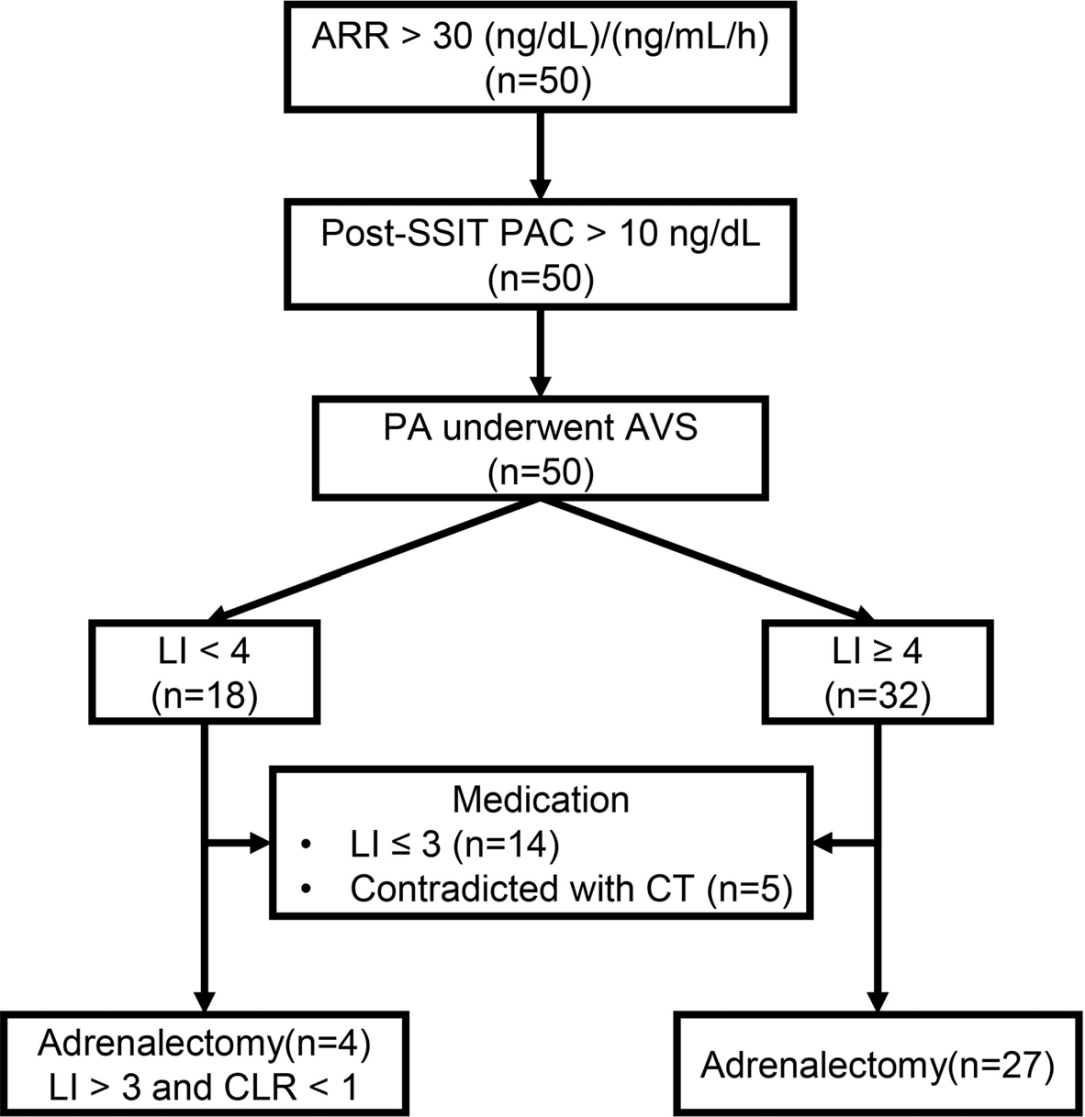
The workup of primary aldosteronism (PA). ARR, Aldosterone-renin-ratio; Post-SSIT, Post-supine saline infusion test; PA, Primary aldosteronism; AVS, Adrenal venous sampling; LI, Lateralization index; CLR, Contralateral ratio; CT, Computed tomography.

A total of 50 patients were enrolled in this study (**Figure 1**), with 32 and 18 patients in h-LI and l-LI groups, respectively. As shown in **Table 1**, there were no significant differences in age, sex, body mass index (BMI), hypertension duration, number of antihypertensive medications, ambulatory BP, and serum potassium levels between the two groups. Compared with the l-LI group, the h-LI group had higher levels of 24 h urinary aldosterone (26.8 ± 11.3 µg/24 h vs 16.7 ± 6.9 µg/24 h, P = 0.0003) and supine PAC (308 (205–423) pg/mL vs. 185 (128-217) pg/mL, P = 0.0003) on admission. In addition, the pre-supine saline infusion test (pre-SSIT) supine PAC (after correction of hypokalemia) in the h-LI group was higher than that in the l-LI group (356 ± 150 pg/mL vs. 251 ± 104 pg/mL, P =0.0114).

**Table 1:** Clinical characteristics of patients stratified by different lateralization index (LI).

Characteristic	All patients (n=50)	LI≥4 (N=32)	LI<4 (N=18)
**Age (years)**	49.5 ± 10.9	49.1 ± 10.1	50.3 ± 12.6
**Male sex (%)**	36 (72.0)	22 (68.8)	14 (77.8)
**BMI (kg/m^2^)**	26.4 ± 3.7	25.7 ± 3.4	27.7 ± 4.0
**Hypertension duration (years)**	10.0 (4.0-20.0)	10.0 (3.5-13.0)	13.0 (5.0-20.0)
**Antihypertensive drugs (n)**	3.0 (2.0-3.0)	3.0 (2.0-3.0)	3.0 (2.0-3.0)
**On admission**			
** 24 h SBP (mm Hg)**	142 ± 13	142 ± 13	140 ± 11
** 24 h DBP (mm Hg)**	89 ± 10	91 ± 9	86 ± 9
** 24 h urinary Na^+^ (µg/24 h)**	158.7 ± 65.5	148.6 ± 63.4	176.6 ± 67.2
** Plasma creatinine (mmol/L)**	74.1 ± 19.9	74.7 ± 22.8	73.0 ± 13.9
** eGFR (mL/min·1.73 m^2^)**	100.9 ± 19.2	99.5 ± 20.7	103.3 ± 16.6
** 24 h urinary protein (mg/24 h)**	153 (123-234)	154 (123-243)	148 (117-221)
** Serum K^+^ (mmol/L)**	3.34 ± 0.30	3.32 ± 0.31	3.38 ± 0.28
** Supine PAC (pg/mL)**	231 (178-367)	308 (205-423) **	185 (128-217)
** Supine PRA (ng/mL·h)**	0.32 (0.16-0.58)	0.32 (0.17-0.62)	0.35 (0.14-0.51)
** Supine ARR ([pg/mL]/[ng/mL·h])**	809 (436-1801)	987 (526-2454)	459 (336-1316)
** 24 h urinary aldosterone (µg/24 h)**	23.2 ± 11.0	26.8 ± 11.3 **	16.7 ± 6.9
**Pre-SSIT**			
** Serum K^+^ (mmol/L)**	3.84 ± 0.32	3.90 ± 0.31	3.76 ± 0.33
** PAC (pg/mL)**	318 ± 143	356 ± 150 *	251 ± 104
** PRA (ng/mL·h)**	0.36 (0.15-0.73)	0.36 (0.17-0.67)	0.40 (0.15-0.87)
** ARR ([pg/mL]/[ng/mL·h])**	749 (338-1669)	806 (473-1915)	521 (230-1326)

Values are indicated as the mean ± standard deviation or as median (25th and 75th).

SBP, systolic blood pressure; DBP, diastolic blood pressure; K^+^, potassium ions; eGFR, estimated glomerular filtration rate (Chronic Kidney Disease Epidemiology Collaboration); PAC, plasma aldosterone concentration; PRA, plasma renin activity; ARR, aldosterone-to-renin ratio; Pre-SSIT, Pre-supine saline infusion test.

*P < 0.05, **P < 0.001 (LI ≥ 4 vs LI<4).

### Characterization of uEVs

**Figure 2:**
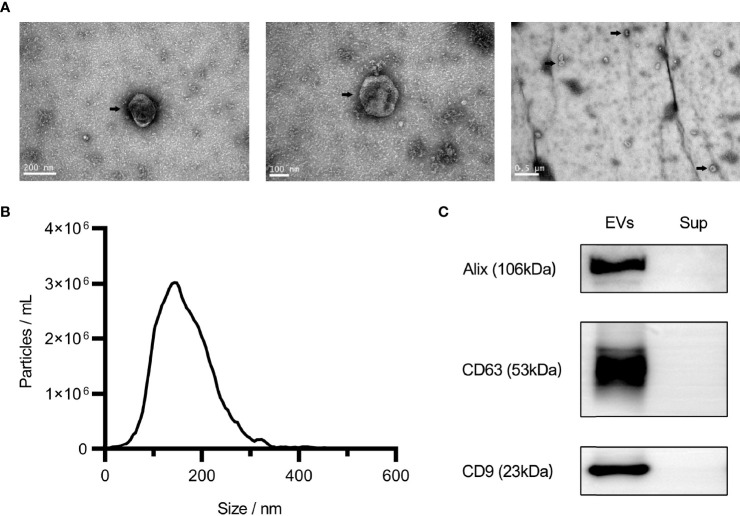
Characteristics of urinary extracellular vesicles (EVs). Electron microscopy analysis of EVs under different magnifications, as shown by the arrow **(A)**. Nanoparticle tracking analysis of EVs **(B)**. Western blot analysis of Alix, CD63, and CD9 **(C)**. Sup, Supernatant.

The results of the characterization of uEVs are shown in **Figure 2**. The particles extracted from urine were characterized using NTA and transmission electron microscopy. The results showed that these particles were cup-shaped in morphology and ranged from 70 to 150 nm in diameter (**Figures 2A, B**). These are two typical characteristics of uEVs. Furthermore, the results of western blotting showed that all uEVs samples were positive for EVs markers (CD9, CD63, and Alix), which were not present in the supernatant (**Figure 2C**).

### NCC and pNCC Expression for Different Lateralization Indices

**Figure 3:**
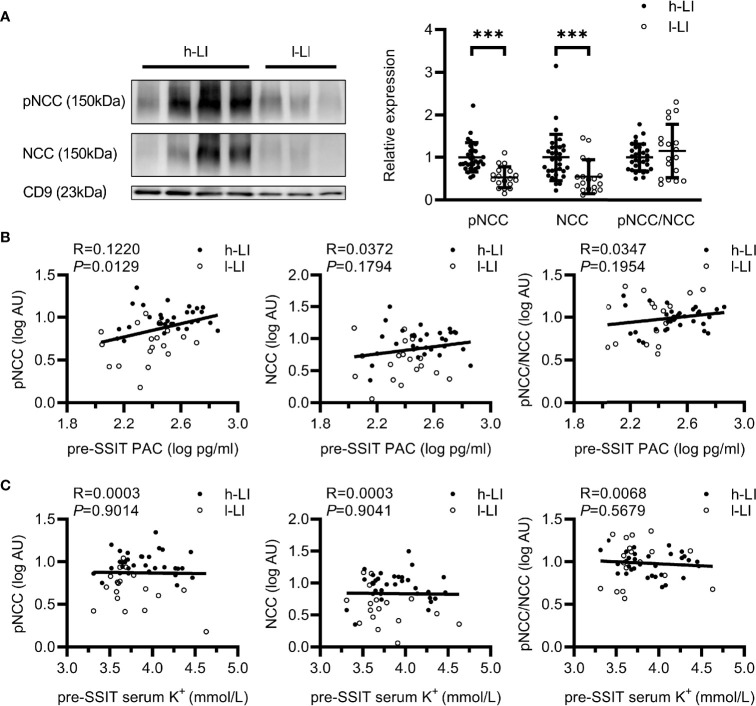
Expression of renal sodium transporters in patients with different lateralization index (LI). Western blot analysis of pNCC and NCC in uEVs of patients with PA. Patients with PA were divided into h-LI (n=32) and l-LI (n=18) groups **(A)**. Correlations between pNCC or NCC expression and PAC or serum potassium **(B, C)**. PA, Primary aldosteronism; h-LI, high lateralization index; l-LI, low lateralization index; PAC, plasma aldosterone concentration; pre-SSIT, pre-supine saline infusion test. ***P < 0.001.

Compared with the l-LI group, pNCC and NCC are more abundant in the uEVs of the patients in the h-LI group (P <.0001, P =0.0002, respectively). The abundance of pNCC and NCC in patients of the h-LI group was approximately 1.89 and 1.82 folds higher than those in the l-LI group (**Figure 3A**), respectively. Higher NCC abundance in uEVs was observed in the h-LI group (1.82 folds, P = 0.0002). There was no significant difference in the pNCC/NCC ratio between the two groups. Furthermore, plasma PAC correlated positively with pNCC (R = 0.1220, *P* = 0.0129), but not with NCC (**Figure 3B**). There was no correlation between serum potassium and pNCC or NCC expression (**Figure 3C**).

### NCC and pNCC Expression in Patients With or Without KCNJ5 Somatic Mutation

**Figure 4:**
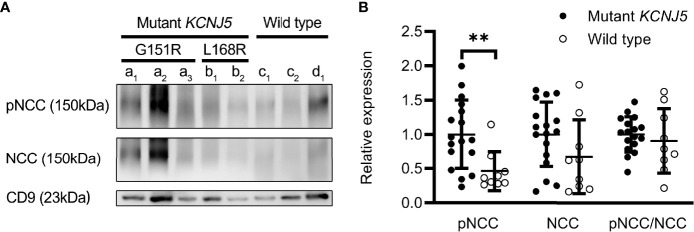
Expression of renal sodium transporters in patients with different genotypes. Western blot analysis of pNCC and NCC in patients with (n=17) or without (n=9) *KCNJ5* mutation **(A)**. Aldosterone-producing adenomas (a–c). G151R mutation on *KCNJ5* (a). L168R mutation on *KCNJ5* (b). No *KCNJ5* mutation (c). Adrenocortical hyperplasia (d). Quantification of western blotting analysis **(B)**. **P < 0.01.

Twenty seven patients underwent unilateral adrenalectomy in our hospital, and four patients received operations in other hospitals (the pathological sections were not obtained). Sanger sequencing was performed for 27 patients. Among them, 26 cases were confirmed as aldosterone-producing adenoma (APA) and one (LI≥ 4) as adrenal hyperplasia (**Table 2**). Somatic KCNJ5 mutations were detected in 17 (65.4%) of the 26 APA cases (**Table 2**), but no mutations were detected in one hyperplasia case. KCNJ5 mutations included G151R and L168R mutations in eleven and five cases, respectively, and an insertion mutation (p.T148_149insW) in one patient. As shown in **Table S1**, at baseline, a trend toward higher level of supine PAC and pre-SSIT PAC in the KCNJ5 mutation group was observed but without statistical significance. Carriers of somatic KCNJ5 mutations, compared with non-carriers, had a higher abundance of pNCC in uEVs (2.16 folds, P=0.0039) (**Figures 4A, B**). There was no significant difference between the two groups in pNCC-to-NCC ratio and NCC abundance.

**Table 2:** Somatic mutation detection.

Genotype	Adrenal adenomas (N=26)	Adrenocortical hyperplasia (N=1)
*KCNJ5* mutation	17	.
*G151R*	11	.
*L168R*	5	.
*T148_149insW*	1	.
*KCNJ5* wild type	9	1

## Discussion

In this study, we investigated whether pNCC and NCC in uEVs, important indicators of true aldosterone status, can be used as biomarkers to distinguish between unilateral and bilateral aldosterone overproduction in patients with PA. The results revealed that pNCC and NCC expression in the h-LI group were higher than those in the l-LI group (1.89 folds and 1.82 folds, respectively). In addition, pNCC content increased in uEVs in patients with a KCNJ5 mutation. This finding indicates that pNCC and NCC could be potential indicators of lateral hyperaldosteronism before AVS.

This is the first study in humans to document the expression of pNCC and NCC in uEVs in two main subtypes of PA. The observation regarding the relationship between pNCC and NCC in uEVs and PAC in this study merits discussion. To the best of our knowledge, early studies suggest that pNCC and NCC in uEVs of humans or animals are possible markers of aldosteronism. Van der Lubbe et al. reported that the infusion of aldosterone in rats increased pNCC abundance by three folds and NCC abundance by 1.5 folds in uEVs, respectively. The pNCC expression in uEVs of patients with PA was 2.6 folds higher than patients with essential hypertension (16). Subsequently, Wolley et al. reported that mineralocorticoid administration in humans increases the levels of NCC and pNCC in uEVs rapidly and sustainably (17). Consistent with the above results, in our study, the abundance of pNCC and NCC in uEVs increased by approximately 1.89 folds and 1.82 folds respectively in the h-LI group, while the simultaneous pre-supine saline infusion test PAC level was 1.42 folds higher than that in the l-LI group. To further support this concept, a 2.16-fold increase in pNCC abundance was observed in patients with *KCNJ5* somatic mutations compared to those without *KCNJ5* mutations. These mutation carriers also had higher PAC levels. Additionally, pNCC, but not NCC, manifested significant positive correlation with plasma PAC level, which suggest that pNCC was a better indicator of true aldosterone status.

The expression and modification of NCC have been attributed to the effects of aldosterone. More recently, several studies in animal and cell models have demonstrated an important role for serum potassium in regulating NCC (12). *In vitro*, high-K^+^ exposure reduces NCC abundance in a ubiquitin-mediated manner in renal cortical tubules; conversely, K^+^ deficiency stimulates NCC via a kinase cascade involving no lysine (WNK) kinases (21, 22). Similarly, low- and high-K^+^ diets can rapidly increase or reduce NCC phosphorylation by changing the serum K^+^ concentration in mice (22). In our study, no correlation was found between NCC or pNCC abundance and serum potassium. Van der Lubbe et al. once reported that upon either high-dose or low-dose (long-term) aldosterone infusion in rats, pNCC expression in uEVs increases, followed by a plateau, suggesting that the expression and activation of NCC by long-term aldosterone infusion will eventually reach saturation (16). The evolution of PA is a relative long way that hypokalemia secondary to hyperaldosteronism only presented in the late phase of PA in around 30% patients (10). When hypokalemia was corrected by KCL supplementation, the level of PAC increased further, as observed in our study. In this context, the expression and activation of NCC in uEVs in patients with PA were mainly affected by long-term pathological aldosterone overproduction instead of hypokalemia, which is secondary to hyperaldosteronism. Recently, Wolley et al. reported that serum potassium levels correlate negatively with NCC and pNCC abundance in uEVs of patients with PA (17). This inconsistency of the effects of serum potassium on NCC may be attributable to different study methods and subjects. In our study, hypokalemia was corrected as much as possible by KCL supplementation before supine saline infusion test, whereas in the study of Wolley’s, no KCL supplementation was performed before fludrocortisone suppression test and not all patients were finally diagnosed to have PA.

Our study reported that the prevalence of KCNJ5 mutations in APAs was 65.4%, which is relatively lower than that reported in our previous study (76.8%) (20). In addition, the abundance of pNCC was higher in mutation carriers, which implied that pNCC in uEVs might be an indicator of KCNJ5 mutations. Recently, several studies have indicated that somatic KCNJ5 mutations are an independent predictor of favorable outcomes after adrenalectomy (5–7, 23). High pNCC expression in uEVs might, therefore, be an indicator of KCNJ5 mutations and AVS.

This study had several limitations. First, the number of patients included in this study was relatively small. These results should be further confirmed in a larger cohort using further quantitative methods such as mass spectrometry. Second, the spot urine sample was collected for the convenience of the patients instead of 24 h urine, which reflects the expression of renal sodium channel protein more comprehensively; however, we corrected it with urinary creatinine. Third, our study did not reflect the changes of pNCC or NCC levels in uEVs after PA treatment. Therefore, further investigations are required to study the expression of pNCC or NCC in uEVs of patients with PA after adrenalectomy or mineralocorticoid antagonist treatment to clarify the impact of different treatment on these uEVs.

## Conclusion

In conclusion, our results revealed that the abundance of pNCC in uEVs was 1.89 folds higher in lateralized aldosteronism and 2.16 folds higher in patients with somatic KCNJ5 mutations. The expression of pNCC in the uEVs may serve as an indicator of AVS and effectively decrease the number of AVS candidates during the workup of PA.

## Data Availability Statement

The original contributions presented in the study are included in the article/**Supplementary Material**. Further inquiries can be directed to the corresponding authors.

## Ethics Statement

The studies involving human participants were reviewed and approved by The Ruijin Hospital Ethics Committee of Shanghai Jiao Tong University School of Medicine. The patients/participants provided their written informed consent to participate in this study.

## Author Contributions

LK performed the experiments, interpreted the data, and drafted the manuscript. XT and YK collected samples and clinical data. LD analyzed and interpreted the pathological and molecular genetic data. JX performed the adrenal venous sampling. JT, PJG, and J-GW contributed to manuscript writing. LZ and WS designed the study and edited the manuscript. All authors contributed to the manuscript and approved the submitted version.

## Funding

This work was supported by a research grant from the Shanghai Bureau of Health and Family Planning (201740079), the Shanghai Commission of Health (20204Y0007), and the Shanghai Commission of Science and Technology (21ZR1454100).

## Conflict of Interest

The authors declare that the research was conducted in the absence of any commercial or financial relationships that could be construed as a potential conflict of interest.

## Publisher’s Note

All claims expressed in this article are solely those of the authors and do not necessarily represent those of their affiliated organizations, or those of the publisher, the editors and the reviewers. Any product that may be evaluated in this article, or claim that may be made by its manufacturer, is not guaranteed or endorsed by the publisher.

## Abbreviations

PA, primary aldosteronism; uEVs, urinary extracellular vesicles; EVs, extracellular vesicles; AVS, adrenal venous sampling; NCC, sodium chloride cotransporter; pre-SSIT, pre-supine saline infusion test; pNCC, phosphorylated sodium chloride cotransporter; h-LI, high lateralization index; l-LI, low lateralization index; PAC, plasma aldosterone concentration.
