# Supplementary material for: The Role of Urinary Extracellular Vesicles Sodium Chloride Cotransporter in Subtyping Primary Aldosteronism
Source: Front Endocrinol (Lausanne). 2022 Apr 4;13:834409. doi: 10.3389/fendo.2022.834409 (PMC9013911; doi:10.3389/fendo.2022.834409)
Supplement: Supplementary file 1 [file Table_1.docx]

**Supplementary digital content files**

**Table S1. Characteristics of patients with or without *KCNJ5* somatic mutation**

| **Characteristic** | **Mutant *KCNJ5***  **(n=17)** | **Wide Type**  **(n=9)** |
| --- | --- | --- |
| **Age (years)** | 45.8 ± 9.8 | 52.1 ± 11.4 |
| **Male sex (%)** | 9 (52.9%) | 7 (77.8%) |
| **BMI (kg/m2)** | 24.5 ± 3.5 | 26.9 ± 2.3 |
| **Hypertension duration (years)** | 8 ± 6 | 11 ± 7 |
| **Antihypertensive drugs (n)** | 3 (2-3) | 3 (2-3) |
| **On Admission** |  |  |
| **24 h SBP (mm Hg)** | 139 ± 12 | 144 ± 14 |
| **24 h DBP (mm Hg)** | 88 ± 9 | 93 ± 9 |
| **24 h urinary Na^+^ (µg/24 h)** | 155.1 ± 64.6 | 146.0 ± 43.9 |
| **Plasma creatinine (mmol/L)** | 63.2 ± 14.5 | 82.0 ± 27.4 |
| **eGFR (mL/min·1.73 m^2^)** | 109.8 ± 13.0 | 95.4 ± 23.8 |
| **24 h urinary protein (mg/24 h)** | 149 (123-164) | 174 (130-267) |
| **Tumor Size (cm)** | 1.47 ± 0.33 | 1.26 ± 0.42 |
| **Serum K^+^ (mmol/L)** | 3.33 ± 0.30 | 3.34 ± 0.35 |
| **Supine PAC (pg/mL)** | 308 (203-408) | 271 (192-384) |
| **Supine PRA (ng/mL·h)** | 0.33 (0.20-0.53) | 0.29 (0.17-0.85) |
| **Supine ARR ([pg/mL]/[ng/mL·h])** | 1155 (631-1883) | 894 (451-1316) |
| **24 h urinary aldosterone (µg/24 h)** | 23.6 ± 12.4 | 23.2 ± 9.4 |
| **Pre-SSIT** |  |  |
| **Serum K^+^ (mmol/L)** | 3.87 ± 0.34 | 3.92 ± 0.31 |
| **PAC (pg/mL)** | 357 ± 181 | 317 ± 83 |
| **PRA (ng/mL·h)** | 0.31 (0.18-0.65) | 0.50 (0.22-0.92) |
| **ARR ([pg/mL]/[ng/mL·h])** | 784 (472-1899) | 642 (474-1324) |

Values are indicated as means ± standard deviations or as medians (25th and 75th).

SBP, systolic blood pressure; DBP, diastolic blood pressure; K^+^, potassium ions; eGFR, estimated glomerular filtration rate (Chronic Kidney Disease Epidemiology Collaboration); PAC, plasma aldosterone concentration; PRA, plasma renin activity; ARR, aldosterone-to-renin ratio; Pre-SSIT, Pre-supine saline infusion test.

**P* < 0.05, ** *P* < 0.01, ****P* < 0.001 ( Mutant *KCNJ5* vs Wide Type)
